# Corneal dystrophies: pathophysiological, genetic, clinical, and therapeutic considerations

**DOI:** 10.22336/rjo.2021.22

**Published:** 2021

**Authors:** Camelia Constantin

**Affiliations:** * “Dr. Carol Davila” Central Military Emergency Hospital, Bucharest, Romania

**Keywords:** corneal dystrophy, genetics, clinic, histology, treatment

## Abstract

Corneal dystrophies represent a group of progressive, genetically transmitted disorders with variable pathological, histological, and clinical manifestations.

Recent ophthalmological examination techniques and genetic investigation methods have brought benefits in the deepening of these conditions. However, many aspects remain unknown, so the results of treatments are unsatisfactory.

The aim of this article was to recall the clinical, genetic, histological and treatment types of these conditions.

## Introduction

Corneal dystrophies represent a group of progressive, bilateral, non-inflammatory, genetically determined disorders, with a period of occurrence in the first to fourth decade, with a higher frequency secondary to recurrent epithelial erosions and visual acuity impairment [**1**].

Most are characterized by various aspects of corneal opacity. Although still partially known, research in recent years has expanded due to new methods of examination, OCT- tomography in optical coherence and confocal microscopy.

## Previous dystrophies (epithelial and Bowman membrane)

The dystrophy of the anterior basal membrane, known as Cogan’s microcystic dystrophy, has the appearance of subepithelial microcysts, in “finger traces”, “spots”, and “geographic map”. It is most common in practice, although little diagnosed due to variable occurrence.

It usually occurs in the 2nd decade of life, but 10% of patients develop recurrent erosions in the 3rd decade. The causes remain controversial, it is believed to occur rather with age, hereditary transmission, in some cases being AD (autosomal dominant), or in relation to the X chromosome [**3**].

Histologically: thickening of the epithelial basal membrane by the deposition of fibrillation proteins, with the appearance of hyperreflective spots, having as manifestations the appearance of corneal opacities highlighted on biomicroscopic examination [**3**].

More recently, at confocal microscopy and with the help of OCT, extracellular deposits with the appearance of finger trace, stain, and map, have been revealed [**7**-**10**].

There are other subtypes of microcystic dystrophies or in the band, where transition zones are observed between the normal epithelium and areas where the cytoplasm is enlarged by abundant fine vacuoles [**4**-**6**]. 

Treatment is the same as recurrent erosions.

**Gelatinous dystrophy**, or familial subepithelial corneal amyloidosis, with autosomal recessive transmission (AR).

Histologically, it is characterized by the accumulation of amyloid in the subepithelial region of the cornea, with the thinning of the epithelial layer, and incomplete destruction of the Bowman membrane, having a gelatinous, white-yellow appearance [**11**-**13**].

It occurs in the first decade of life, with severe photophobia, foreign body sensation, tear, and reduction of visual acuity. In the advanced stages, neovascularization occurs in the deep stroma and under the epithelium [**3**-**8**].

Surgical treatment is effective only temporarily, as recurrences appear on the graft after a few years. 

## Meesmann epithelial corneal dystrophy 

Transmission: is AD, but also AR, with mutation on gene K3 and K12 [**3**].

Bilateral damage is very rare.

Symptomatology occurs in the first two years of life manifested by eye irritation. 

The biomicroscopic examination shows intraepithelial microcysts, uniform in size, round or oval, with variable density, reaching the maximum in the central area, but they never extend to the limb.

Generally, visual acuity is not affected.

Histologically: irregular thickening of the epithelium with numerous cytoplasmic vacuoles like cysts [**1**]. Cysts contain cellular detritus and intracellular substances of unknown composition [**9**-**14**].

Treatment: it is not necessary because visual acuity is not affected.

## Stromal dystrophies

***Reis-Buckler corneal dystrophy***

Transmission: AD.

The onset of the disease occurs in the first years of life, with moderate impairment of visual acuity. With age, dense and irregular ring opacities appear “in the map” in the Bowman membrane. After the second decade of life, patients no longer experience pain, due to reduced corneal sensitivity [**3**].

Histologically: trapezoidal deposits in the Bowman layer and between epithelial cells.

Treatment: lamellar keratoplasty, but with the high and rapid incidence of the recurrence on the graft [**1**]. 

***Avellino corneal dystrophy***

Transmission: AD, chromosome 5q [**1**]. It is assumed that the pathogenesis is related to autophagy, which is why the mechanism is still less known.

It is known as type II granular corneal dystrophy, which also includes lattice corneal dystrophy. Clinical manifestations combine the characteristics of the two types of dystrophies with granular and lattice opacifications.

It appears in the first and second decade of life.

Granular opacifications occur more frequently than lattice ones, which lead to variable impairment of visual acuity, the location and severity of the disease being observed over time [**3**]. 

Histologically: hyaline granular deposits, superficially located, as well as lattice deposits of amyloid, located deeply [**3**]. 

Depending on the appearance, the lesions are divided into 3 subtypes: type 1, diffuse deposits are located superficially, irregularly; type 2, granular deposits are divided into round surface deposits (type 2 a) and spicules (type 2 b); type 3 a - deposits with short branches, type 3 b - with long branches [**3**].

Treatment: not necessary.

***Central cloudy dystrophy of Francois***

Transmission: possible AD, the detailed mechanism is not known.

The biomicroscopic examination reveals large areas of the posterior stroma and smaller areas of the anterior stroma with polygonal grey opacities, bounded by straight lines, giving the appearance of “crocodile skin” [**3**].

The epithelium and corneal endothelium are not affected. 

Opacities located both centrally and in the periphery of the deep stroma cornea, known as “crocodile skin”, are considered corneal degeneration in relation to age. The difference between the two entities is the mode of transmission.

Histologically: extracellular vacuoles with fibrillation substances [**3**].

Opacities occur through the accumulation of mucopolysaccharides and lipids.

***Schnyder crystalline corneal dystrophy***

Transmission: AD, mutations located on gene 1p34.1-p36, the mechanism remaining uncertain [**1**].

The onset occurs in the second decade of life, with impaired visual acuity when viewed [**1**,**2**].

It is characterized by the appearance of lipid springs and/ or crystalline deposits of cholesterol in the stroma.

Histologically: deposits of lipids and cholesterol in the stroma. In confocal microscopy, superficial epithelial cells are within normal limits, while the basal cell layer is poorly viewed, and shows crystalline deposits with extension to the anterior stroma [**1**,**2**].

Treatment: excimer laser keratectomy [**1**].

***Cogan’s microcystic dystrophy***

Transmission: is the only human disease associated with the mutation of the decorin gene, a small leucine, rich in proteoglycans, which contributes to corneal transparency and refractory stability.

Clinically, manifestations occur shortly after birth and progress with age. Some patients have strabismus or nystagmus.

Biomicroscopic examination: diffuse, bilateral, whitish opacities, like “snowflakes”, in the stroma.

Histologically: epithelial cells are normal in confocal microscopy, but increase the reflectivity of the stroma. The stromal lamellar structure is interrupted, more in the anterior and posterior central stroma.

OCT highlights diffuse reflectivity accentuated in the stroma.

Treatment: in most patients with penetrating keratoplasty performed during youth the results are good.

***Fleck corneal dystrophy***

Transmission: AD, the PIP5K3 gene is responsible for intracellular accumulation [**3**].

Biomicroscopic examination: fine opacities, round-oval, flattened, white-yellow, bilateral, in deep stroma. No systemic anomalies were identified.

The onset occurs in the first years of life, with slow progression, visual acuity not being affected.

Histologically: some keratocytes have fibroglandular tissue in intracytoplasmic vacuoles, afterwards containing lipids and mucopolysaccharides [**3**].

Treatment: not necessary in most cases. No graft rejections were reported 10 years after penetrating keratoplasty.

***Groenouw type I corneal dystrophy***

Belongs to the group RBCD, Thiel-Behnke corneal dystrophy, LCD, Groenouw type II corneal dystrophy.

Transmission: AD, mutation on the TGFBI gene [**3**].

Biomicroscopic examination: stromal opacities, irregular, glassy-looking, or “flakes” [**2**, **3**].

Most patients are asymptomatic, some may develop recurrent erosions. Injuries increase over time, when visual acuity is affected [**3**]. 

Histologically: deposits of phospholipids and microfibrils in keratocytes and epithelial cells. Injuries mostly affect the stroma.

Treatment: keratoplasty in the 6th decade or later. 

***Lattice corneal dystrophy (LCD)***

It is a stromal dystrophy, with the appearance of “ramifications”.

Transmission: AD, with mutation on the TGFI gene, which encodes keratoepitelin, responsible for cellular adhesion [**3**].

It is classified into 3 subtypes: LCD I, LCD II, LCD III, LCD III A [**1**].

Changes occur in the first and second decade of life and progress over time.

Opacities appear in the anterior stroma with linear appearance, recurrent erosions, and “haze” in the center of the anterior stroma, accentuated with age. 

LCD I and LCD II are associated with systemic amyloidosis type V (Meretoja syndrome), transmitted AD [**3**].

LCD II occurs in youth and affects the cornea, skin, and cranial nerves.

LCD III is manifested by the presence of thickened lines in the cornea, stretched from limb to the limb. It is transmitted AR. Recurrent erosions occur rarely, in the 4th decade of life [**3**].

LCD III A has almost the same changes, except for the fact that there are recurrent erosions, and the transmission is AD [**1**-**3**].

Histologically: LCD I is affected both at the stroma and epithelium levels. LCD II is affected at the level of the amyloid deposits under the Bowman layer and sometimes on the epithelial basal membrane. LCD III is affected at the level of the amyloid deposits. Endothelium and Descemet membrane are not affected.

***Macular corneal dystrophy (MCD)***

Transmission: AR, occurs very rarely [**3**].

Biomicroscopic examination: white-grey opacities in the center of the cornea, with age the lesions extend to the periphery and affect the entire stroma. The feature is also thinning the cornea.

Opacities and abnormal structures of the cornea cause severe impairment of visual acuity.

It is classified into 2 subtypes, I and II, defined by the absence or presence of serum sulphate keratin [**3**].

Histologically: accumulation of extracellular deposits in the stroma and Descemet membrane, as well as intracellular storage in keratocyte and endothelium.

Treatment: perforating keratoplasty, with frequent recurrence on the graft [**1**].

***Pre-Descement corneal dystrophy (PDCD) or filiform dystrophy***

Transmission: seems to be related to age, but pathology remains uncertain [**3**].

It appears in the 4th and 6th decade of life.

Biomicroscopic examination: fine opacities in the posterior stroma, representing lipid deposits.

Patients are usually asymptomatic, and visual acuity is rarely affected.

Histopathologically: pathological changes are limited to keratocytes in the posterior stroma, which accumulate lipid materials, more similar lipofuscin, a degenerative pigment that accumulates in aging cells [**2**,**3**].

Treatment: not necessary.

## Posterior corneal dystrophies

***Congenital hereditary endothelial dystrophy (CHED)***

Transmission: AD-CHED 1, AR-CHED 2 [**3**]. 

CHED 1 occurs less frequently, has a clinical location like posterior polymorphic dystrophy (PPMD), while CHED 2 is more severe and occurs more frequently [**3**].

They show up right after birth.

They are characterized by corneal opacification and nystagmus. Damage to the corneal endothelium produces edema, especially stromal, giving the appearance of “glass”. They are bilateral and symmetrical [**1**].

Histopathologically: thickening of the Descemet membrane and endothelial atrophy, amyloid deposits, and spheroidal degeneration.

Differential diagnosis is made with other causes of corneal opacification in newborns: congenital glaucoma, mucopolysaccharides, trauma at birth, keratitis from rubella, sclerocornea.

Treatment: penetrating keratoplasty with chances of success the earlier it is done [**1**].

***Fuchs endothelial corneal dystrophy (FECD)***

It is the most common type of endothelial, bilateral, non-inflammatory, progressive dystrophy, manifested by decreased visual acuity.

Transmission: AD, but many cases are rare [**3**].

Signs: appearance “guttata” in the cornea and “metal beaten”, stromal edema, which gives the so-called “in fog” vision, microcystic epithelial edema, persistent, when the stroma thickens by 30% and bullous keratosis appears, and afterwards degenerative pannus. 

Primary defects are endothelial degenerations, secondary - corneal edema.

Associated manifestations: increased corneal nerve sensitivity, stromal opacification, recurrent corneal erosion, open-angle glaucoma, greater impairment in women, familial predisposition [**3**].

Histologically: fibroblast-like endothelial cells, increase in the number of cytoplasmic filaments, phagocytized pigment granules, increase in the number of hemidesmosomes [**3**].

Treatment: Artificial tears or solution NaCl 5% drops, ointment for maintaining tear film, therapeutic contact lens for comfort and protection, perforating keratoplasty, other options: conjunctival flap, amniotic membrane coverage [**1**,**2**].

***Posterior polymorphous corneal dystrophy (PPCD)***

It is rare, asymptomatic, with damage to the Descemet membrane and endothelium.

Transmission: AD [**1**]. 

Signs: asymmetrical spots, clustered vesicles, bands, geographical gray areas, endothelium like-epithelium (loss of inhibition of contact with proliferation and growth over angle and iris). Some patients develop stromal edema. Moreover, the iris and pupil change like those in iridocorneal syndrome and glaucoma [**1**].

Symptoms: pain, foreign body sensation, tear, photophobia, decreased visual acuity [**1**].

Histologically: blisters, lesions in the band and diffuse opacity.

Treatment: not necessary [**1**].

## Conclusions

Although some corneal dystrophies occur in relation to age, most are associated with genetic mutations.

The most common symptom is decreased visual acuity. Severity is different, in some patients, blindness occurs with age and requires keratoplasty, in others visual acuity remains stable for most of life.

Common signs are loss of vision, foreign body sensation, tear, photophobia, recurrent erosions, or rarer, such as strabismus, nystagmus, glaucoma.

Advanced genetic techniques have allowed genetic mutations to be associated with some dystrophies, but the detailed mechanisms remain uncertain.

For this reason, there is no effective method of treatment. Keratoplasty remains the last possibility of improving visual acuity in severe forms, but with a high relapse rate in a few years.

Because genetic mutations play a role in most dystrophies, studies will contribute greatly to understanding and establishing an effective method of treatment. 

Recently, in-vitro gene therapy seems to have brought new perspectives in the treatment of these conditions.

**Conflict of Interest**

The author declares no conflict of interest.

**Acknowledgements**

None.

**Sources of Funding**

None.

**Disclosures**

None. 
